# Impedance shaping based stabilization control method for DC Micro-grid Feed-forward compensation

**DOI:** 10.1038/s41598-025-14523-w

**Published:** 2025-08-08

**Authors:** Xiaojuan Zhang, Bo Jing, Yuan Wang, Xiangzhen Meng, Jie Zhang

**Affiliations:** 1 College of Mechanical Engineering, Xijing University, Xi’an, China; 2 College of Aeronautical Engineering, Air Force Engineering University, Xi’an, China; 3 School of Electrical and Control Engineering, Shaanxi University of Science and Technology, Xi’an, China

**Keywords:** Constant power unit, Negative impedance characteristics, Feed-forward compensation, Impedance shaping, Stability, Electrical and electronic engineering, Energy grids and networks

## Abstract

The ports of the bi-directional converter exhibit negative impedance characteristics when the energy storage unit of a DC microgrid is operating in charging mode. This can decrease the system’s stability margin, potentially leading to oscillation instability. To address this issue, we propose a feed-forward compensation control method based on impedance shaping. This involves designing a transfer function within the current feed-forward loop of the energy storage converter to ensure that the current reference value follows changes in the bus voltage. Selecting an appropriate time constant ensures that the port impedance of the energy storage unit exhibits a positive resistive characteristic close to the oscillation frequency while retaining a negative impedance characteristic in the low-frequency range. This ensures the stability of system operation. MATLAB/Simulink simulations and RT-LAB semi-physical platform testing have verified that the proposed control method effectively enhances the system’s stability margin, ensuring stable operation and minimal steady-state voltage deviation.

## Introduction

 The advantages of high efficiency, modularity, and flexible control of DC micro-grids, making them widely applied in various fields such as renewable energy integration, electric vehicle charging, and power systems of data centers^1–4^. However, a large number of closed-loop controlled power electronic converters exhibit negative impedance characteristics externally, which are prone to interacting with the equivalent LC loops formed in the system and triggering system oscillations^5–8^.

The stability analysis method based on the system impedance is more common. It analyzes the system as a whole from the source and load perspectives, using the ratio of the output impedance to the input impedance at the DC bus port as the loop gain. If the Nyquist curve of the system loop gain does not include the (−1, j0) point, it is judged to be a stable system^9–11^. At present, there are two main types of methods for suppressing system oscillations: passive damping methods and active damping methods^12,13^. The passive damping method aims to enhance the system’s stability margin by adding actual passive devices. However, these methods result in system losses and are deemed inefficient^14,15^. Active damping methods can effectively avoid the aforementioned shortcomings, prompting numerous scholars to conduct in-depth research on this approach. Zhuang proposed an active damping control method for load converters to address the LC filter resonance issue caused by constant power loads. By implementing a virtual RC network in parallel at the load side to reshape the input impedance characteristics, it improves the system phase margin by 30° and suppresses resonance peaks by 20dB^16^. Zhang proposes an approach, which connects a virtual impedance in parallel or series with the input impedance of the load converter, it corrects the magnitude and phase of the input impedance and solves the instability problem of the cascaded system^17^. References [16–17] discuss active damping added to the “load” converter. While it can effectively suppress oscillations, it also alters the impedance characteristics of the constant power load, negatively impacting its performance. Guan proposes a dual-current active damping control strategy based on the inverter-side current and grid-connected current feedback to enhance the resonance stability of the grid-connected converter. This strategy involves incorporating active damping in the current feedback loop to enhance the system’s ability to suppress resonance effects^18^. Zeng proposed a novel bilateral active damping control strategy based on the feedbacks of both DC capacitor voltage and AC filter capacitor current. The capacitor current of the LCL filter is used as feedback to realize the zero pole cancellation of the transfer function, reduce the order of system equivalent model, track the migration of the resonant point, and suppress the resonant peak^19^. Zhu Establishes a droop-adaptive virtual impedance control method for grid-forming converters. By dynamically coupling virtual resistance compensation with droop coefficients, it achieves significant THD reduction in grid-side converters^20^. Hosseinipour proposes an active damping control method based on virtual RC parallel impedance, which helps enhance the stability margin of the system^21^. Oshnoei presents utilizes a neural network based intelligent control algorithm to adaptively adjust the damping coefficient in the virtual impedance. This approach overcomes the control system’s reliance on operating point conditions^22^.

Different forms of active damping methods, as discussed in references^18–22^, are implemented on the “source” converter. Among them, the active damping compensation discussed in references^18–21^ affects the port impedance across the entire frequency range, and the regulation of the DC bus voltage may degrade under heavy load conditions of the system. In contrast, reference^22^ uses an intelligent algorithm to adaptively adjust the damping coefficient, thereby minimizing the impact of virtual impedance on the impedance characteristics of the system in other frequency bands. Reference^23^ analyzed the electrolytic capacitor inverter. By adding a capacitor voltage difference term to the output of the current controller, a control strategy was proposed to provide leading phase compensation for the DC bus voltage and input current of the electrolytic capacitor inverter to suppress LC resonance. The proposed electrolytic capacitor inverter can operate in both motor mode and regenerative mode. Reference^24^proposes an active power control method for a three-phase capacitorless PWM converter operating in minimum switching mode. In the proposed method, the motor-side inverter regulates the DC bus voltage to an efficiency-optimised level, and the grid-side converter always operates within the boundary of the limit hexagon in the voltage vector space. This minimises the number of switching operations of the grid-side converter and improves system efficiency. However, the implementation is more complex. The references above solely address the oscillation issue resulting from the impedance mismatch between the constant power load and the preamplifier circuit.

However, none of the existing methods take into account the instability risks brought about by the constant power characteristics of the energy storage device during constant current charging.

To address the aforementioned issues, this paper proposes a feed-forward compensation control method based on impedance shaping and applies it to the energy storage converter. The main contributions of this paper lie in the following aspects:

1) The reference current of the current control loop is corrected by multiplying the feed-forward compensation transfer function. Adjusting the time constant in this function causes the constant power unit to simulate a positive resistance characteristic near the oscillation frequency while maintaining the original negative impedance characteristic in the low-frequency band.

2) The small signal analysis shows that the proposed control method can shape the port impedance of the energy storage unit. This shaping not only converts the negative resistance of the energy storage unit input to positive during charging, but also enhances the phase of the energy storage unit output impedance during discharging. This enhancement results in the system having a better phase margin in both charging and discharging modes.

3) Analyzed the influence of changes in controller parameters and time constants on the stability of the system, and further confirmed the accuracy of the theoretical analysis through simulation and experimental results.

 The structure of this paper is as follows: In Sect. "System architecture and modeling", the structure of the DC micro-grid system was analyzed, and the bidirectional DC-DC converter and the closed-loop control system were modeled. In Sect. "Feedforward Compensation Control Based On Impedance Shaping", a feed-forward compensation control method based on impedance shaping is proposed, including the instantaneous power correction method for the constant power unit and the feed-forward compensation control method based on impedance shaping. In Sect. "Stability Analysis", the feed-forward compensation effects and system stability under charging and discharging states are compared respectively, and the influence of changes in controller parameters and time constants on system stability is analyzed. In Sect. "Simulation And Experimental Verifications", the accuracy of the theoretical analysis is further confirmed by simulation and experiment results. In Sect. "Conclusion", the conclusion of the whole paper is presented.

## System architecture and modeling

**Fig. 1:**
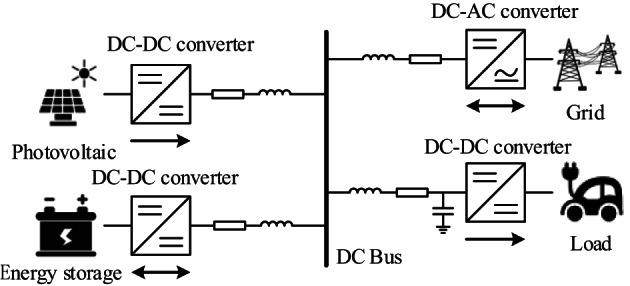
Structure of DC micro-grid

The structure of a typical DC micro-grid system is shown in Fig. 1. It consists of an energy storage system, a photovoltaic power generation system, a grid system, and a load. The common load is connected through a Buck converter. Line impedance exists in each unit on the connecting line with the DC bus. The input side of the load converter is connected to a bus capacitor to filter out voltage ripples.

In island operation mode, a DC micro-grid disconnects from the grid system and relies on energy storage to balance supply and demand. When the power generated by the PV system is insufficient, both the energy storage and PV system supply power to maintain a constant load. During this period, the load behaves like a constant-power load, exhibiting negative impedance characteristics at the port. This can lead to instability in the system due to impedance mismatch between the front-stage converter, line impedance, and filter capacitance of the low damping LC loop. When there is abundant photovoltaic power generation and the system load is small, the energy storage system receives power from the photovoltaic source with constant power. During this period, the energy storage system acts like a constant power unit, resulting in a negative impedance at the port. This impedance may also be mismatched with the impedance of the low-damped LC loop, which consists of the front-stage converter, line impedance, and bus-side capacitance, posing a risk of instability.

### Modeling of Bi-directional DC-DC converter for energy storage

**Fig. 2:**
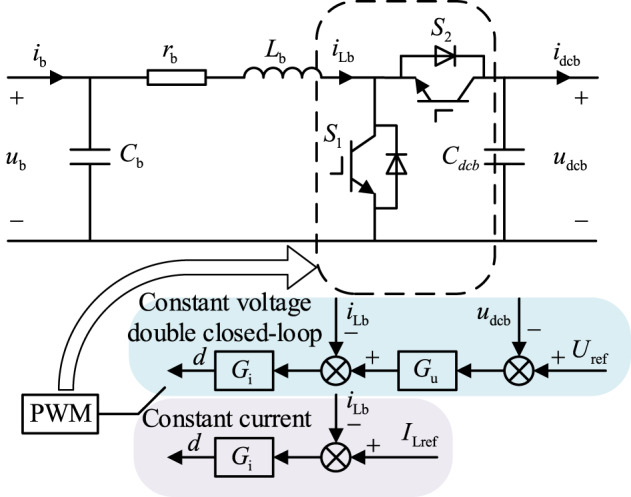
Energy storage converter topology and its control structure

The energy storage bi-directional DC-DC converter operates in Boost mode during discharging and Buck mode during charging. The circuit topology and control structure are shown in Fig. 2 below. In the figure, *C*_b_ represents the battery-side capacitance; *C*_dcb_ represents the bus-side capacitance; *L*_b_ represents the filtering inductor; *r*_b_ represents the inductor parasitic resistance; *U*_ref_ and *I*_Lref_ represent the reference voltage and current values for constant voltage double closed-loop control and constant-current control, respectively; *u*_dcb_ and *i*_Lb_ represent the actual output voltage of the converter and the inductor current, respectively, and *G*_u_ and *G*_i_ represent the transfer functions of voltage and current controllers. The small-signal state equations are as follows:

1 $$\left\{ \begin{gathered} sL_{\rm b}{\hat{i}}_{\rm Lb}+r_{\rm b}{{\hat {i}}_{\rm Lb}={\hat {u}}_{\rm b} } - (1 - D){{\hat {u}}_{\rm dcb}}+{U_{\rm dcb}}\hat {d} \hfill \\ s{C_{\rm{dcb} }}{{\hat {u}}_{\rm{dcb} }}=(1 - D){{\hat {i}}_{\rm Lb }} - {I_{\rm Lb}}\hat {d} - {{\hat {i}}_{\rm dcb}} \hfill \\ {{\hat {i}}_{\rm b}}={{\hat {i}}_{\rm{Lb} }}+s{C_{\rm b}}{{\hat {u}}_{\rm b}} \hfill \\ \end{gathered} \right.$$

Where ${\hat {i}_{{\text{Lb}}}}$ represents the inductor current perturbation; ${\hat {u}_{\text{b}}}$ represents the input voltage perturbation; ${\hat {u}_{{\text{dcb}}}}$ represents the output voltage perturbation of the energy storage converter; ${\hat {i}_{\text{b}}}$ represents the input current perturbation; f represents the duty-cycle signal perturbation; *D* represents the duty-cycle signal direct flow; *U*_dcb_ and *I*_Lb_ represent the output voltage of the converter and the direct flow of the inductor current, respectively.

### Closed-loop control model

**Fig. 3:**
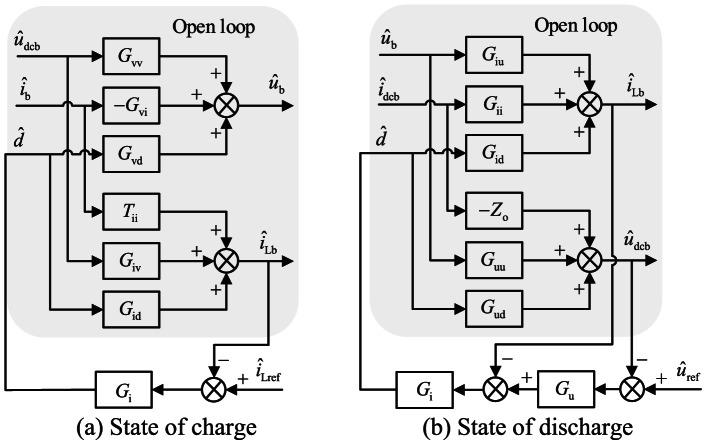
Small signal model of energy storage converter

The control structure of the bi-directional DC-DC energy storage converter under closed-loop control is shown in Fig. 2. The energy storage is controlled by a single current closed-loop control during charging and a constant-voltage double closed-loop control during discharging. From the control structure in Fig. 2, the closed-loop small-signal model under the two control methods is shown in Fig. 3.

The figure ${\hat {u}_{{\text{ref}}}}$ represents the perturbation quantity of the output voltage reference value, and the corresponding transfer function between each perturbation quantity is shown in the Fig. 3.

The bus-side port impedance for both control modes can be obtained as follows:

2 $$\begin{gathered} \frac{1}{{{Z_{inc}}}}={Y_{inc}}=D \times {\left. {\frac{{{{\hat {i}}_{\operatorname{Lb} }}}}{{{{\hat {u}}_{\operatorname{dcb} }}}}} \right|_{{{\hat {i}}_b}=0,{{\hat {i}}_{\operatorname{Lref} }}=0}}+{I_L} \times {\left. {\frac{{\hat {d}}}{{{{\hat {u}}_{dcb}}}}} \right|_{{{\hat {i}}_b}=0,{{\hat {i}}_{\operatorname{Lref} }}=0}} \\ =\frac{{D{G_{iv}} - {I_{Lb}}{G_{iv}}{G_i}}}{{1+{G_i}{G_{id}}}} \\ \end{gathered}$$

3 $${Z_{oc}}= - {\left. {\frac{{{{\hat {u}}_{dcb}}}}{{{{\hat {i}}_{dcb}}}}} \right|_{{{\hat {u}}_b}=0,{{\hat {u}}_{ref}}=0}}=\frac{{{Z_o}(1+{G_i}{G_{\operatorname{id} }})+{G_{ii}}{G_i}{G_{ud}}}}{{1+{G_i}{G_{id}}+{G_u}{G_i}{G_{ud}}}}$$

Where *Z*_inc_ represents the closed-loop input impedance of the bus side of the converter operating in charging mode; *Y*_inc_ represents the closed-loop input impedance of the bus side of the converter operating in charging mode; *Z*_oc_ represents the closed-loop output impedance of the bus side of the converter operating in discharging mode.

## Feedforward compensation control based on impedance shaping

### Instantaneous power correction method for constant power units

The low-damped LC loop equivalent in the DC micro-grid system interacts with the constant power unit, leading to a mismatch in input and output impedances. Consequently, the system experiences oscillatory instability with an oscillatory angular frequency as follows:

4 $${\omega _{{\text{res}}}}=2{\pi}{f_{{\text{res}}}} \approx \frac{1}{{\sqrt {{L_{{\text{eq}}}}{C_{{\text{eq}}}}} }}$$

Where *L*_eq_ and *C*_eq_ represent the equivalent inductance and equivalent capacitance of the low-damping LC link, respectively; *f*_res_ represents the oscillation frequency.

**Fig. 4:**
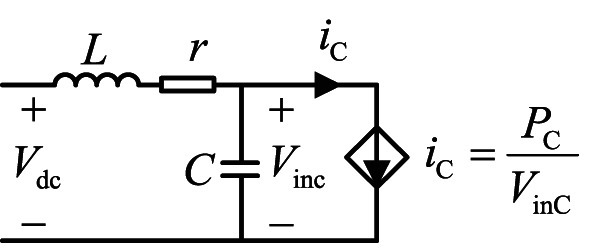
Equivalent model of a constant power unit

**Fig. 5:**
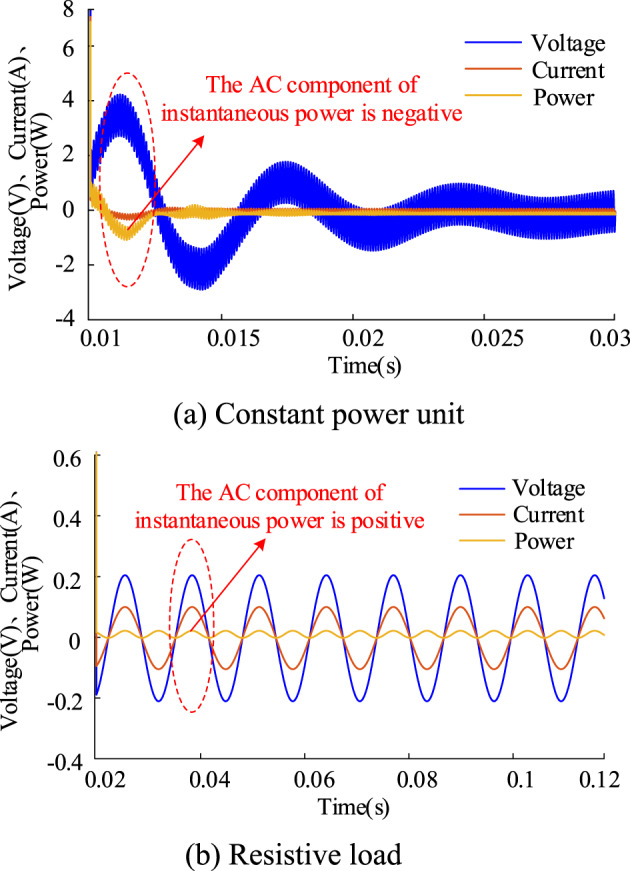
Waveform of AC components of voltage, current and instantaneous power

The power obtained by the constant power unit from the DC bus is instantaneous and constant. Therefore, it can be equated to a current source, as shown in Fig. 4 below. Here, *i*_C_ represents the input current of the constant power unit, *V*_inC_ represents the input voltage of the constant power unit, and *P*_C_ represents the instantaneous power value of the constant power unit. The DC component of *P*_C_ is always positive, indicating that the constant power unit is consuming power. However, the AC component of *P*_C_ is negative, as shown in Fig. 5, due to the negative impedance characteristic of the constant power unit. In contrast, the voltage and current of a purely resistive load are always in phase, resulting in positive power. This is because the constant power unit exhibits negative impedance characteristics, causing the voltage and current to be in opposite phases. In contrast, the voltage and current of a purely resistive load are always in phase, and the power is always positive. The negative impedance of the constant power unit can easily lead to impedance mismatch with the LC loop, reducing the stability margin of the system and exacerbating system oscillations.

Combined with the theory mentioned above, if the PC can exhibit a power characteristic similar to that of a pure resistive load near the oscillation frequency, meaning its AC small-signal component behaves positively, this will align the voltage and current phases of the constant-power unit as closely as possible. This alignment will reduce the original negative impedance characteristic and enhance the stability margin of the system. Therefore, the PC is modified as follows, and the modified instantaneous power value of the constant power unit is $P_{{\text{C}}}^{*}$

5 $$P_{{\text{C}}}^{*}=K(s) \times {V_{{\text{inC}}}}$$

Where *K*(s) represents the compensation transfer function.

*K*(s) is selected to ensure that $P_{{\text{C}}}^{*}$ follows *V*_inC_ only near the oscillation frequency, while still maintaining the original constant power characteristic in the low-frequency range. In other words, $P_{{\text{C}}}^{*}$ is independent of *V*_inC_ when the power is still *P*_C_. From this, *K*(s) is determined as follows:

6 $$\begin{array}{*{20}{c}} {K(s)=\frac{{{P_{\text{C}}}}}{{{V_{{\text{inC}}}}}} \times \frac{1}{{1+s\tau }}}&{\forall P_{{\text{C}}}^{*}=K(s)} \end{array} \times {V_{{\text{inC}}}}$$

7 $$P_{{\text{C}}}^{*}=\frac{{{P_{\text{C}}}}}{{{V_{{\text{inC}}}}}} \times \frac{1}{{1+s\tau }} \times {V_{{\text{inC}}}}$$

Where *τ* represents the time constant of the first-order low-pass filter.

By selecting an appropriate *τ* value, the port characteristics of the constant power unit exhibit a positive resistance characteristic near the oscillation frequency to improve the system’s stability margin. This is achieved while retaining the original negative impedance characteristics in the low-frequency band, ensuring that the system can effectively regulate the bus voltage without experiencing significant voltage drops.

### Feed-forward compensation control method based on impedance shaping

In order to prevent the constant power unit from interacting with the equivalent low-damping LC loop in the system and decreasing the system’s stability margin, the reference current value *I*_Lref_ is adjusted analogously to the correction method of Eq. (7) for power. This adjustment ensures that the system maintains voltage-current phase consistency around the oscillating frequency and demonstrates a positive resistive characteristic. The resulting corrected reference current value is as follows:

8 $$I_{{{\text{Lref}}}}^{*}=\left( {\frac{{{I_{{\text{Lref}}}}}}{{{U_{{\text{dcb}}}}}} \times \frac{1}{{1+s\tau }}} \right) \times {U_{{\text{dcb}}}}$$

Let

9 $$K(s)=\frac{{{I_{{\text{Lref}}}}}}{{{U_{{\text{dcb}}}}}} \times \frac{1}{{1+s\tau }}$$

In order to determine an appropriate value for the time constant to enable impedance shaping at the oscillation frequency, the expansion in Eq. (9) is represented as a combination of the DC component and the AC component through small-signal analysis as follows:

10 $$I_{{{\text{Lref0}}}}^{{\text{*}}}+\hat {i}_{{{\text{Lref}}}}^{{\text{*}}}=\left( {\frac{{{I_{{\text{Lref0}}}}+{{\hat {i}}_{{\text{Lref}}}}}}{{{U_{{\text{dcb0}}}}+{{\hat {u}}_{{\text{dcb}}}}}} \times \frac{1}{{1+s\tau }}} \right) \times ({U_{{\text{dcb0}}}}+{\hat {u}_{{\text{dcb}}}})$$

Where the subscript “0” represents the DC component and “ˆ” represents the AC small signal perturbation.

For single-current closed-loop control, we can set ${\hat {i}_{{\text{Lref}}}}=0$ and disregard the higher-order terms in the formula to get

11 $$I_{{{\text{Lref0}}}}^{*}+\hat {i}_{{{\text{Lref}}}}^{{\text{*}}}=\left( {\frac{{{I_{{\text{Lref0}}}}}}{{{U_{{\text{dcb0}}}}}}\left( {1 - \frac{{{{\hat {u}}_{{\text{dcb}}}}}}{{{U_{{\text{dcb0}}}}}}} \right) \times \frac{1}{{1+s\tau }}} \right) \times \left( {{U_{{\text{dcb0}}}}+{{\hat {u}}_{{\text{dcb}}}}} \right)$$

Since $\frac{{{I_{{\text{Lref0}}}}}}{{{U_{{\text{dcb0}}}}}} \times \frac{1}{{1+s\tau }}=\frac{{{I_{{\text{Lref0}}}}}}{{{U_{{\text{dcb0}}}}}}$, it follows that

12 $$\begin{gathered} I_{{{\text{Lref0}}}}^{*}+\hat {i}_{{{\text{Lref}}}}^{*}=\left( {\frac{{{I_{\operatorname{Lref} 0}}}}{{{U_{{\text{dcb0}}}}}} - \frac{{{I_{{\text{Lref0}}}}}}{{U_{{{\text{dcb0}}}}^{2}}} \times {{\hat {u}}_{{\text{dcb}}}} \times \frac{1}{{1+s\tau }}} \right) \times \left( {{U_{{\text{dcb0}}}}+{{\hat {u}}_{{\text{dcb}}}}} \right) \\ ={I_{{\text{Lref0}}}}+\frac{{{I_{{\text{Lref0}}}}}}{{{U_{{\text{dcb0}}}}}}{{\hat {u}}_{{\text{dcb}}}} - \frac{{{I_{{\text{Lref0}}}}}}{{U_{{{\text{dcb0}}}}^{{\text{2}}}}}{{\hat {u}}_{{\text{dcb}}}} \times \frac{1}{{1+s\tau }} \times {U_{{\text{dcb0}}}} \\ - \frac{{{I_{{\text{Lref0}}}}}}{{U_{{{\text{dcb0}}}}^{{\text{2}}}}}{{\hat {u}}_{{\text{dcb}}}} \times \frac{1}{{1+s\tau }} \times {{\hat {u}}_{{\text{dcb}}}} \\ \end{gathered}$$

In the steady state case $I_{{{\text{Lref0}}}}^{{\text{*}}}={I_{{\text{Lref0}}}}$, we get

13 $$\hat {i}_{{{\text{Lref}}}}^{{\text{*}}}=\frac{{{I_{{\text{Lref0}}}}}}{{{U_{{\text{dcb0}}}}}} \times \left( {1 - \frac{1}{{1+s\tau }}} \right) \times {\hat {u}_{{\text{dcb}}}}=F(s){\hat {u}_{{\text{dcb}}}}$$

That is

14 $$F(s)=\frac{{{I_{{\text{Lref0}}}}}}{{{U_{{\text{dcb0}}}}}} \times \left( {1 - \frac{1}{{1+s\tau }}} \right)$$

To ensure that the system adapts to changes in the oscillation frequency, *F*(s) needs to be straight flow near the oscillation frequency, where the break frequency of *F*(s) is 1/*τ*. The expression for *F*(s) at frequency much smaller than the break frequency is:

15 $$F(s) \approx \frac{{{I_{{\text{Lref0}}}}}}{{{U_{{\text{dcb0}}}}}} \times s\tau$$

When the frequency is much larger than the break frequency *F*(s), a direct flow occurs with the expression:

16 $$F(s) \approx \frac{{{I_{{\text{Lref0}}}}}}{{{U_{{\text{dcb0}}}}}}$$

We observed that when the system’s oscillation frequency is higher than the break frequency, it can ensure a direct flow in the vicinity of the oscillation frequency *F*(s). This causes $\hat {i}_{{{\text{Lref}}}}^{*}$ follow the change of ${\hat {u}_{{\text{dcb}}}}$ at the oscillation frequency, exhibiting a similar positive resistance characteristic to that of a resistive load. This is beneficial for the stable operation of the system.

For constant voltage double closed-loop control, the current reference is generated by the voltage controller with a perturbation that, similar to the above derivation process, yields.

17 $$\begin{gathered} \hat {i}_{{{\text{Lref}}}}^{*}=\frac{{{I_{{\text{Lref0}}}}}}{{{U_{{\text{dcb0}}}}}} \times \left( {1 - \frac{1}{{1+s\tau }}} \right) \times {{\hat {u}}_{{\text{dcb}}}}+\frac{1}{{1+s\tau }} \times {{\hat {i}}_{{\text{Lref}}}} \\ =F(s){{\hat {u}}_{{\text{dcb}}}}+H(s){{\hat {i}}_{{\text{Lref}}}} \\ \end{gathered}$$

That is

18 $$F(s)=\frac{{{I_{{\text{Lref0}}}}}}{{{U_{{\text{dcb0}}}}}} \times \left( {1 - \frac{1}{{1+s\tau }}} \right)$$

19 $$H(s)=\frac{1}{{1+s\tau }}$$

Let ${\hat {u}_{{\text{ref}}}}=0$, we get

20 $${\hat {i}_{{\text{Lref}}}}= - {G_{\text{u}}}{\hat {u}_{{\text{dcb}}}}$$

Since

21 $$\hat {i}_{{{\text{Lref}}}}^{*}=\left( {\frac{{s\tau }}{{1+s\tau }} - \frac{1}{{1+s\tau }} \times \frac{{{k_{{\text{pu}}}}s+{k_{{\text{iu}}}}}}{s}} \right){\hat {u}_{{\text{dcb}}}}=T(s){\hat {u}_{{\text{dcb}}}}$$

To ensure that system $\hat {i}_{{{\text{Lref}}}}^{*}$ follows ${\hat {u}_{{\text{dcb}}}}$ at the oscillation frequency, *T*(s) needs to exhibit a linear behavior near the oscillation frequency and a break frequency of $\sqrt {{{{k_{{\text{iu}}}}} \mathord{\left/ {\vphantom {{{k_{{\text{iu}}}}} \tau }} \right. \kern-0pt} \tau }}$. At frequency significantly lower than the break frequency, *T*(s) displays an integral characteristic, while at frequency much higher than the break frequency, *T*(s) behaves linearly and shows a positive resistive characteristic. Therefore, when the oscillation frequency is greater than the break frequency, it can be guaranteed that *T*(s) represents a straight flow near the oscillation frequency.

**Fig. 6:**
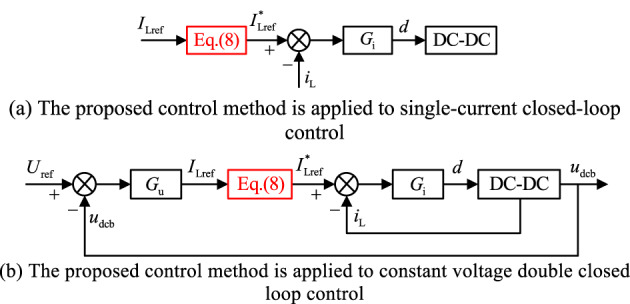
Structure diagram of energy storage converter applying the proposed control

The principal block diagram of the proposed feed-forward compensation control method applied to single current closed-loop control as well as constant voltage double closed-loop control is shown in Fig. 6.

## Stability analysis

### Feed-forward compensation effectiveness

**Fig. 7:**
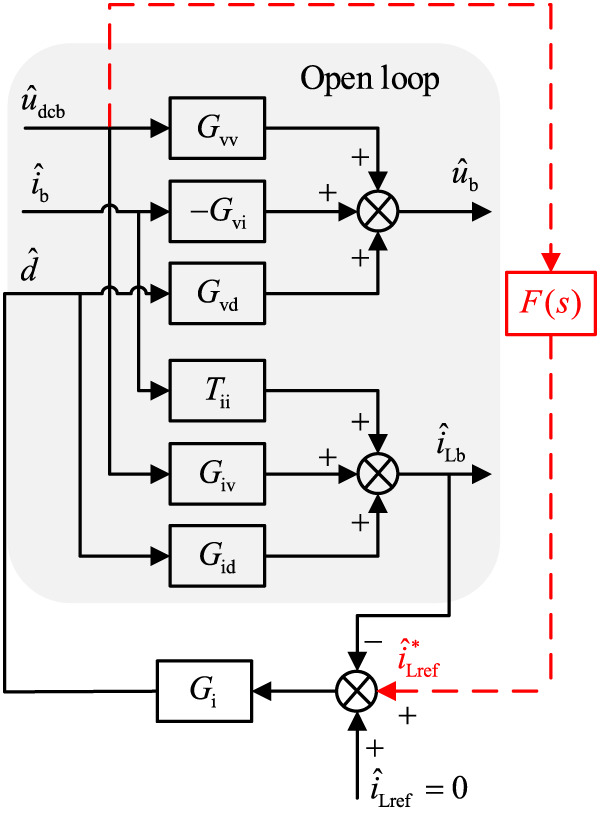
Small signal model of single current closed-loop control with feed-forward compensation

When the energy storage system is in the charging state, the small-signal model of the single-current closed-loop control after the introduction of feed-forward compensation is shown in Fig. 7.

**Fig. 8:**
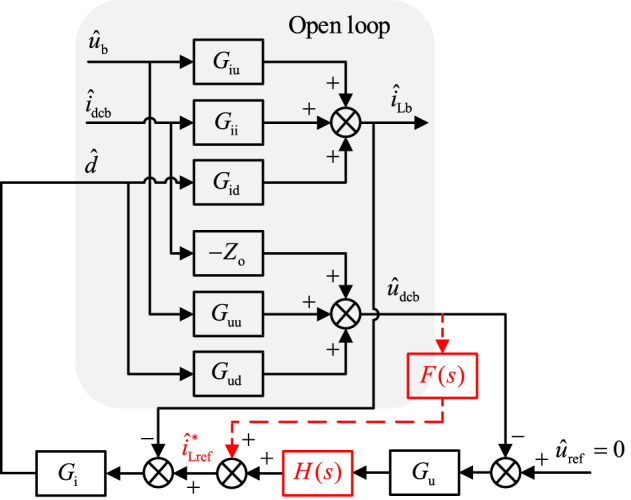
Small signal model of constant voltage double closed-loop control with feed-forward compensation

According to Fig. 8, the input conductance of the energy storage unit after adding feed-forward compensation can be determined by setting${\hat {i}_{\text{b}}}=0$.

22 $$\begin{gathered} Y_{{{\text{inc}}}}^{\prime }=D \times {\left. {\frac{{{{\hat {i}}_{{\text{Lb}}}}}}{{{{\hat {u}}_{{\text{dcb}}}}}}} \right|_{{{\hat {i}}_{\text{b}}}=0,{{\hat {i}}_{{\text{Lref}}}}=0}}+{I_{\text{L}}} \times {\left. {\frac{{\hat {d}}}{{{{\hat {u}}_{{\text{dcb}}}}}}} \right|_{{{\hat {i}}_{\text{b}}}=0,{{\hat {i}}_{{\text{Lref}}}}=0}} \\ =\frac{{D{G_{{\text{iv}}}}+DF(s){G_{\text{i}}}{G_{{\text{id}}}} - {I_{\text{L}}}{G_{\text{i}}}{G_{{\text{iv}}}}+{I_{\text{L}}}F(s){G_{\text{i}}}}}{{1+{G_{\text{i}}}{G_{{\text{id}}}}}} \\ \end{gathered}$$

Input conductance without feed-forward compensation is as follows:

23 $${Y_{{\text{ori}}}}={Y_{{\text{inc}}}}$$

After adding feed-forward compensation, the input conductance can be expressed as follows:

24 $${1 \mathord{\left/ {\vphantom {1 {Z_{{{\text{inc}}}}^{\prime }=}}} \right. \kern-0pt} {Z_{{{\text{inc}}}}^{\prime }=}}Y_{{{\text{inc}}}}^{\prime }={Y_{{\text{ori}}}}+{Y_{{\text{vir}}}}$$

Where

25 $${Y_{{\text{vir}}}}=\left( {\frac{{{I_{\text{L}}}+D{G_{{\text{id}}}}}}{{1+{G_{\text{i}}}{G_{{\text{id}}}}}}} \right)F(s){G_{\text{i}}}$$

Where $Z_{{{\text{inc}}}}^{\prime }$ represents the closed-loop input impedance of the energy storage system after feed-forward compensation is added; $Y_{{{\text{inc}}}}^{\prime }$ represents the closed-loop input conductance of the energy storage system after feed-forward compensation is added; *Y*_vir_ represents the virtual conductance added by the equivalent of feed-forward compensation in the energy storage system, which is expressed as the virtual impedance in the form of ${Z_{{\text{vir}}}}={1 \mathord{\left/ {\vphantom {1 {{Y_{{\text{vir}}}}}}} \right. \kern-0pt} {{Y_{{\text{vir}}}}}}$. By adding virtual impedance, impedance shaping is achieved on the original input impedance of the energy storage system, enhancing the negative impedance characteristic of the energy storage system.

When the energy storage system is in the discharged state, the small signal model of the constant voltage double closed-loop control after introducing feed-forward compensation is shown in Fig. 8.

According to Fig. 8, the output impedance of the energy storage unit after adding feed-forward compensation is as follows:

26 $$\begin{gathered} Z_{{{\text{oc}}}}^{{{\prime }}}= - {\left. {\frac{{{{\hat {u}}_{{\text{dcb}}}}}}{{{{\hat {i}}_{{\text{dcb}}}}}}} \right|_{{{\hat {u}}_{\text{b}}}=0,{{\hat {u}}_{{\text{ref}}}}=0}} \\ =\frac{{{Z_{\text{o}}}(1+{G_{\text{i}}}{G_{{\text{id}}}})+{G_{{\text{ii}}}}{G_{\text{i}}}{G_{{\text{ud}}}}}}{{1+{G_{\text{i}}}{G_{{\text{id}}}}+{G_{\text{u}}}H(s){G_{\text{i}}}{G_{{\text{ud}}}} - {G_{\text{i}}}{G_{{\text{ud}}}}F(s)}} \\ \end{gathered}$$

It follows that the virtual conductance added by the feed-forward compensation equivalent in the energy storage system is as follows:

27 $${Y_{{\text{vir}}}}= - \frac{{{G_{\text{i}}}{G_{{\text{ud}}}}F(s)}}{{{Z_{\text{o}}}(1+{G_{\text{i}}}{G_{{\text{id}}}})+{G_{{\text{ii}}}}{G_{\text{i}}}{G_{{\text{ud}}}}}}$$

**Fig. 9:**
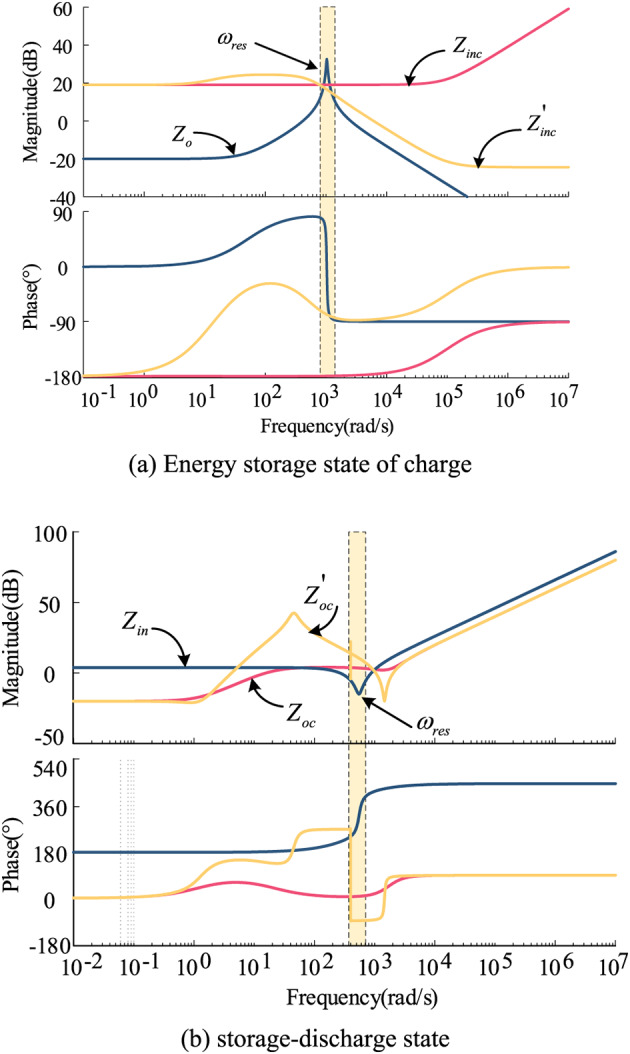
Bode diagram comparing the input impedance and output impedance of the system with and without feed-forward compensation.

Based on the fact that the energy storage system operates in two states of charging and discharging, the Bode plots of the port impedance with and without the introduction of feed-forward compensation are plotted, as shown in Fig. 9.

**Fig. 10:**
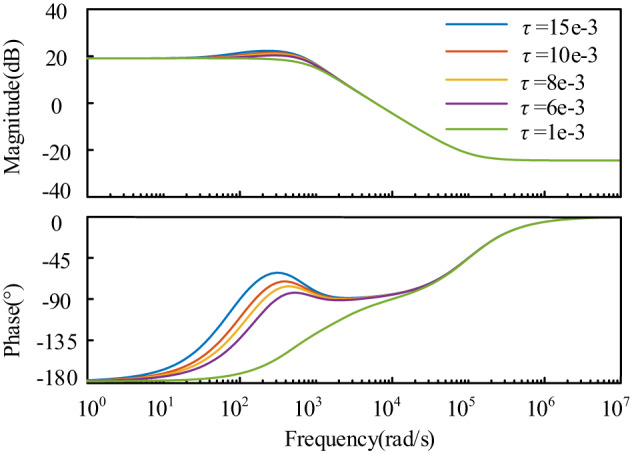
Bode plot of input impedance corresponding to different time constants.

As depicted in Fig. 9(a), after implementing feed-forward compensation in the energy storage charging state, the impedance phase is situated in the fourth quadrant near the oscillation frequency (between 0° and − 90°), indicating a positive resistive impact. This configuration enhances the shaping of the input impedance, leading to an improved phase margin of the system and promoting stable system operation. Simultaneously, the low-frequency band maintains a negative resistive attribute, thereby avoiding significant steady-state voltage deviations. In Fig. 9(b), the introduction of feed-forward compensation also enhances the phase of the output impedance of the energy storage unit near the oscillation frequency in the discharge state. The phase difference between the two impedances is reduced, thereby improving the stability margin of the system. The specific results of the system’s input impedance and phase enhancement corresponding to different time constants are shown in Fig. 10; Table 1.

**Table 1:** Phase increase in input impedance at the Oscillation frequency under different time constants

τ	Phase at oscillation frequency	Degree of phase enhancement	Stability
*τ* = 1e-3	−135°	45°	Unsteady
*τ* = 6e-3	−84.5°	95.5°	Steady
*τ* = 8e-3	−80.6°	99.4°	Steady
*τ* = 10e-3	−77.6°	102.4°	Steady
*τ* = 15e-3	−74°	106°	Steady

From Fig. 10, it is evident that as the time constant increases, the phase enhancement effect becomes more pronounced. Additionally, the positive resistive component of the phase of the input impedance at the oscillation frequency (closer to the real axis) also increases. From Table 1, when the time constant *τ* = 1e-3, the phase of the input impedance at the oscillation frequency is −135°, which still exhibits negative resistance characteristics, leading to system instability. To achieve stabilization, the time constant needs to meet specific conditions where the phase at the oscillation frequency shows positive resistance characteristics, ensuring system stability is maintained.

**Fig. 11:**
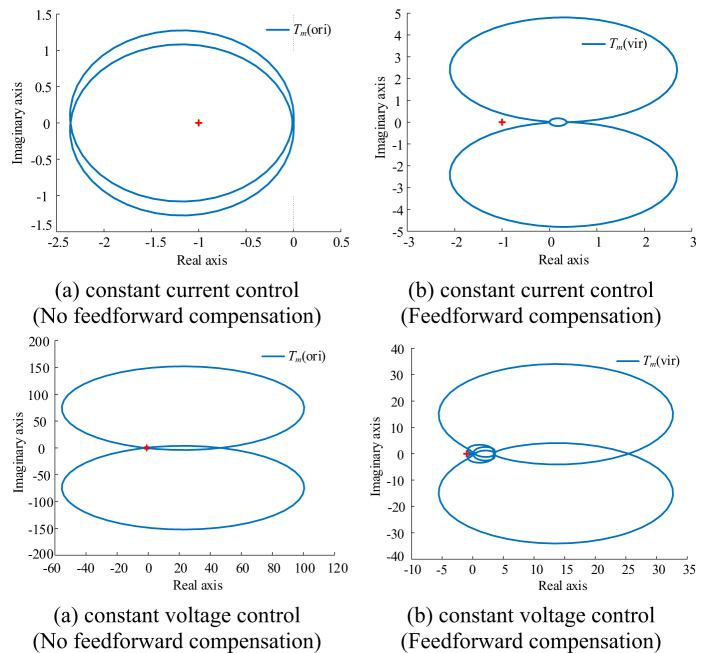
Nyquist curve of system loop gain *T*_m_

Figure 11 shows the Nyquist curve of the system loop gain with and without feed-forward compensation when the energy storage system is in two states: charging and discharging. The loop gain transitions from the original encircled (−1, j0) point to the unencircled (−1, j0) point with the introduction of feed-forward compensation. This transition demonstrates that feed-forward compensation can stabilize the system under both control modes, highlighting the effectiveness of this compensation method.

### Controller parameters

**Fig. 12:**
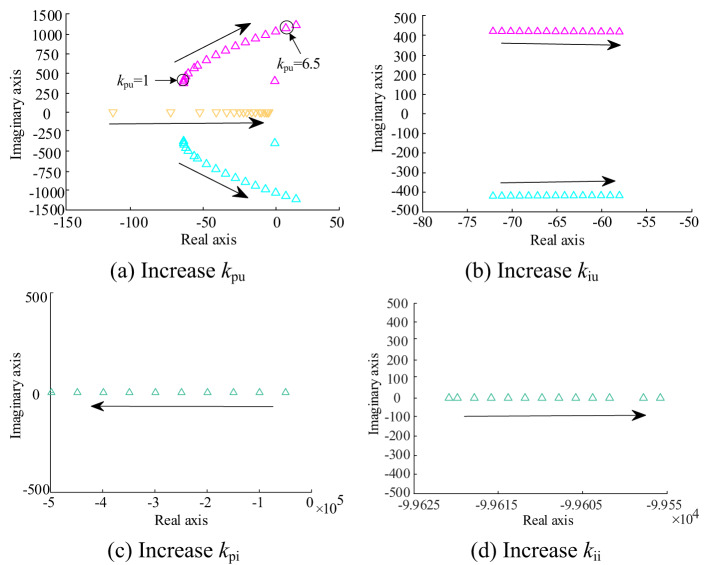
Pole distribution of the system

In order to discuss the influence of the controller parameters on the stability of the system, the distribution of the system poles with the variation of the controller parameters is plotted, as shown in Fig. 12. The impact of varying the voltage controller scaling factor on the distribution of the system poles is shown in Fig. 12(a). The figure displays three poles that are notably influenced by the variation of the voltage controller scaling factor, denoted as *k*_pu_ (ranging from 0.2 to 7). Among these poles, two form a pair of conjugate poles, while the third pole stands alone. This pair of conjugate poles gradually moves towards the imaginary axis as *k*_pu_ increases. When kpu reaches 6.5, this pair of conjugate poles enters the right half-plane of the s-plane, rendering the system unstable. At the same time, there is an inflection point in the trajectory of this pair of poles with respect to *k*_pu_, at which the distance between the poles and the imaginary axis is the largest, and the degree of stability is also the highest. This inflection point corresponds to a *k*_pu_ of 1, which represents the optimal proportionality system value. The other single pole also moves towards the imaginary axis as *k*_pu_ increases, but the magnitude of this change decreases as *k*_pu_ increases. This suggests that it is becoming less affected by changes in *k*_pu_.

The effect of varying the integration coefficient *k*_iu_ of the voltage controller on the distribution of the system poles is shown in Fig. 12(b). It can be observed that the pair of conjugate poles depicted in the figure shift towards the imaginary axis as *k*_iu_ increases (ranging from 2 to 30). The degree of influence of the variation of *k*_iu_ on the pole positions is not as significant as that of *k*_pu_. The poles remain located in the left half-plane of the s-plane when the variation of *k*_iu_ is within a larger range, and the system can still maintain stability. *k*_iu_ is chosen to be 20.

The distribution of the system poles changes with the variation of the current controller scaling factor *k*_pi_, as shown in Fig. 12(c). As *k*_pi_ increases (ranging from 0.5 to 5), one of the negative real poles of the system moves away from the imaginary axis, enhancing stability. The value of *k*_pi_ is chosen to be 1.

The distribution of the system poles changes as the current controller integration coefficient *k*_ii_ is shown in Fig. 12(d). A negative real pole of the system shifts towards the imaginary axis as *k*_ii_ increases (ranging from 5 to 30), leading to a gradual decrease in stability. Similarly, the variation of the current controller *k*_pi_ has a significant effect on the stability of the system compared to *k*_ii_. The value of *k*_ii_ is chosen to be 5.

### Time constants

**Fig. 13:**
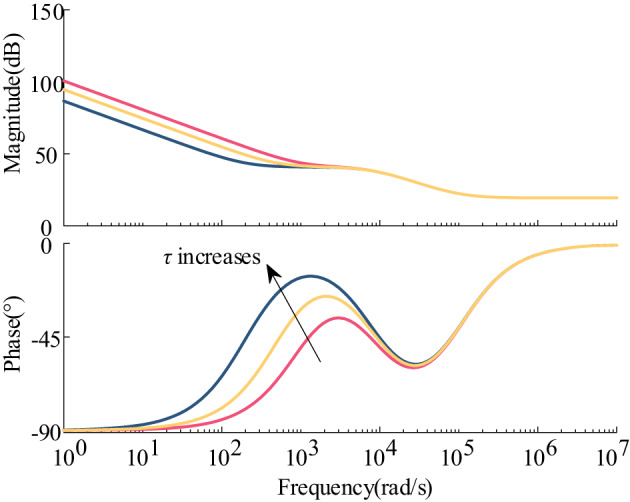
Bode diagram of the equivalent added virtual impedance

The stability margin of the system can be enhanced by implementing feed-forward compensation in the control of the energy storage converter, as shown in Fig. 13. The equivalent virtual impedance is added after the introduction of feed-forward compensation under the single-current closed-loop control. It is evident that the virtual impedance enhances the phase of the impedance as the time constant *τ* increases. The effect of improving the − 180° phase of the constant power unit in the original system becomes increasingly apparent. Consequently, the stability margin of the system will be continuously enhanced. From Fig. 13, it can be seen that the phase of the added virtual impedance ranges between 0° and − 90°, which can be likened to the RC parallel branch of the resistor-parallel capacitor.

**Fig. 14:**
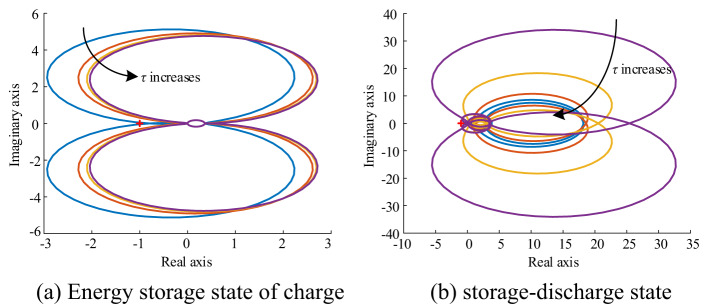
Nyquist curve of system loop gain for different *τ*

Figure 14 shows the Nyquist curves of the overall loop gain of the system when the energy storage system is operating in charging and discharging states, respectively. According to Fig. 14(a) and (b), the Nyquist curves gradually move away from the point (−1, j0) as the time constant increases. This results in an improvement in the stability margin of the system, consistent with the analysis of the Bode plot shown in Fig. 13.

## Simulation and experimental verifications

### Simulation verification

In order to verify the effectiveness of the proposed control method, a simulation model shown in Fig. 1 is constructed in the MATLAB/Simulink environment. The switching frequency is set at 10 kHz, the bus voltage is 600 V, the battery voltage is 400 V, and the output voltage of the load side is 200 V. The structural parameters of each unit in the micro-grid system and the control parameters are shown in Table 2.

**Table 2:** Parameters of each unit in the system

Microgrid units	Parameters	Parameter value
Photovoltaic unit	Photovoltaic Side Capacitance *C*_pv_	380$\mu$F
	Inductor *L*_pv_/*r*_pv_	6mH/0.01Ω
	Bus-side capacitance *C*_dcpv_	470$\mu$F
	*k*_pu_/*k*_iu_	1/10
	*k*_iu_/*k*_ii_	1/5
Energy storage units	Energy storage side capacitance *C*_b_	380$\mu$F
	Inductor *L*_b_/*r*_b_	5mH/0.01Ω
	Bus-side capacitance *C*_dcb_	470$\mu$F
	*k*_pu_/*k*_iu_	1/20
	*k*_iu_/*k*_ii_	1/5
Load unit	Load Side Capacitance *C*_L_	470$\mu$F
	Inductor *L*_L_/*r*_L_	6mH/0.01Ω
	*k*_pu_/*k*_iu_	5/20
Line	Line Resistance *r*	1Ω
	Line Inductor *L*	2mH
	Filter capacitor *C*	2500$\mu$F

**Fig. 15:**
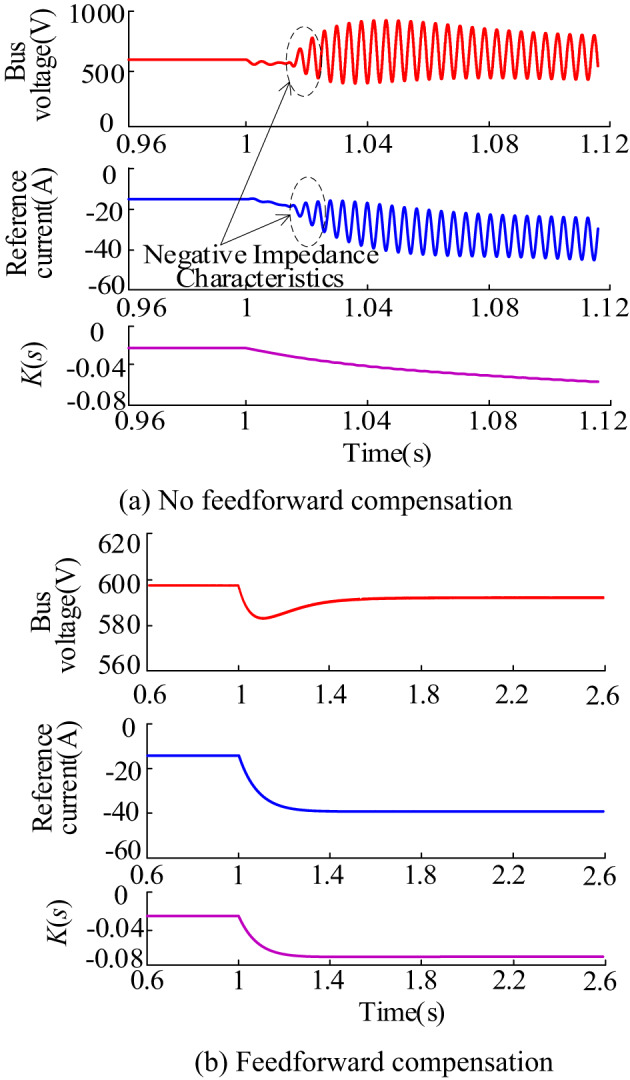
Waveform of bus voltage, reference current, and *K*(s)

When the energy storage is operating in the charging state, the absorbed power of the energy storage system abruptly changes from 6 kW to 16 kW at t = 1s. The waveforms of the bus voltage, current reference value, and K(s) with and without feed-forward compensation are shown in Fig. 15. Figure 15(a) depicts the scenario without feed-forward compensation. In this case, the bus voltage and current reference values exhibit oscillatory behavior after a sudden change, with opposite phase changes during the oscillation process, demonstrating negative impedance characteristics. Figure 15(b) illustrates the situation with added feed-forward compensation. In this scenario, the oscillatory behavior of the bus voltage and current reference values after a sudden change is more effectively suppressed, and the phase alignment between voltage and current is significantly improved. Tends to be consistent. The waveform of *K*(s) is close to direct current (DC) due to the presence of a low-pass filter in *K*(s).

**Fig. 16:**
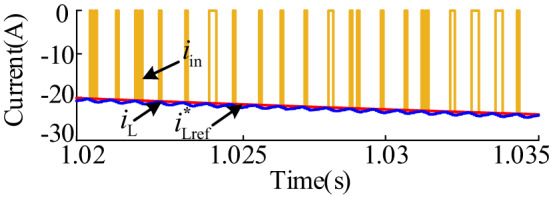
${i_{in}}$、${i_L}$、$\operatorname{I} _{{{\text{Lref}}}}^{{\text{*}}}$ waveforms during charging

Figure 16 shows the waveforms of the input current *i*_in_, inductor current *i*_L_ and current reference value of the energy storage system under the specified operating conditions. At this time, the energy storage converter operates in Buck mode. Therefore, the input current is not continuous; instead, the input current and inductor current follow the variation of the current reference value.

**Fig. 17:**
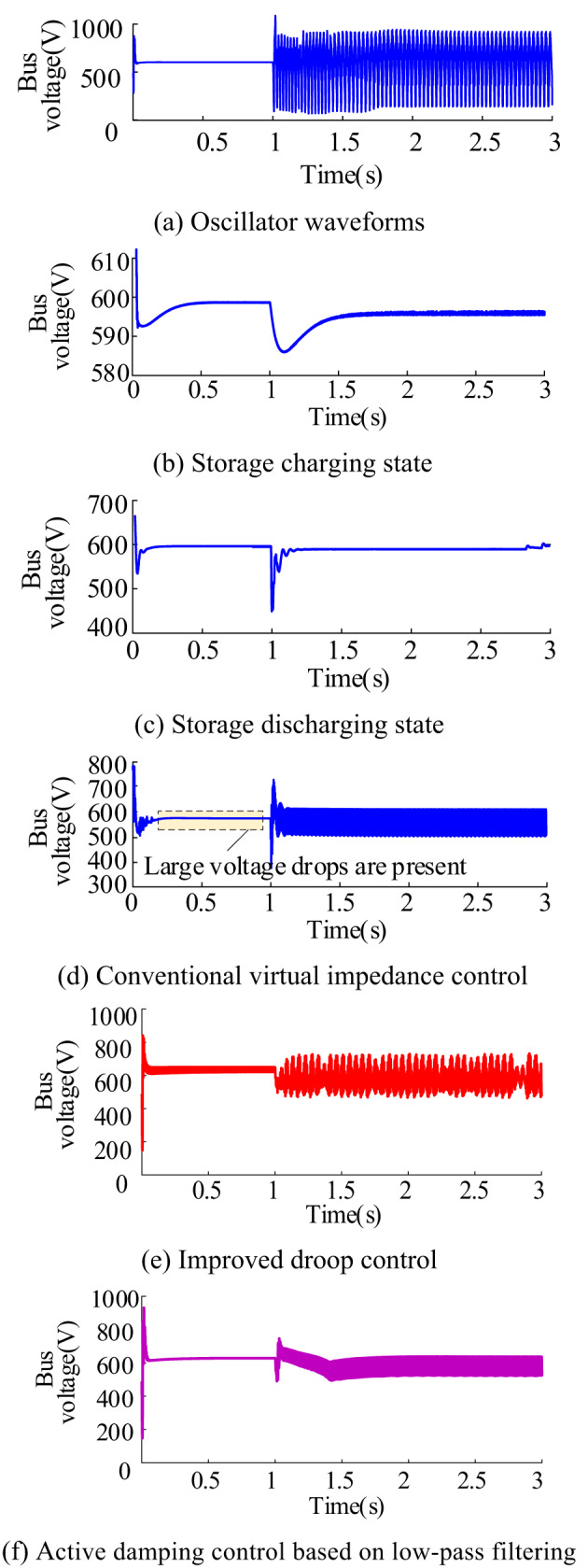
Bus voltage waveforms under various conditions

In order to verify the effectiveness of the feed-forward compensation control method for the two states of energy storage system charging and discharging, the simulation of bus voltage waveforms in response to power changes was conducted. As depicted in Fig. 17(a)- (c), Fig. 17(a) illustrates the bus voltage oscillation phenomenon that occurs in the system following a sudden increase in power. Figure 17(b) displays the bus voltage waveforms during the charging state of the energy storage system. In this scenario, the absorbed power of the energy storage system abruptly rises from 6 kW to 16 kW at t = 1s. After integrating the feed-forward compensation, it is evident that the control method proposed in this study effectively mitigates oscillations, resulting in minimal voltage fluctuation. In the bus voltage waveform, it can be seen that the control method proposed in this paper can better inhibit oscillation, resulting in smaller voltage fluctuations. Figure 17(c) illustrates the bus voltage waveforms during the energy storage and discharge state. In this scenario, the absorbed power of the load rises from 20 kW to 50 kW at t = 1s.

**Fig. 18:**
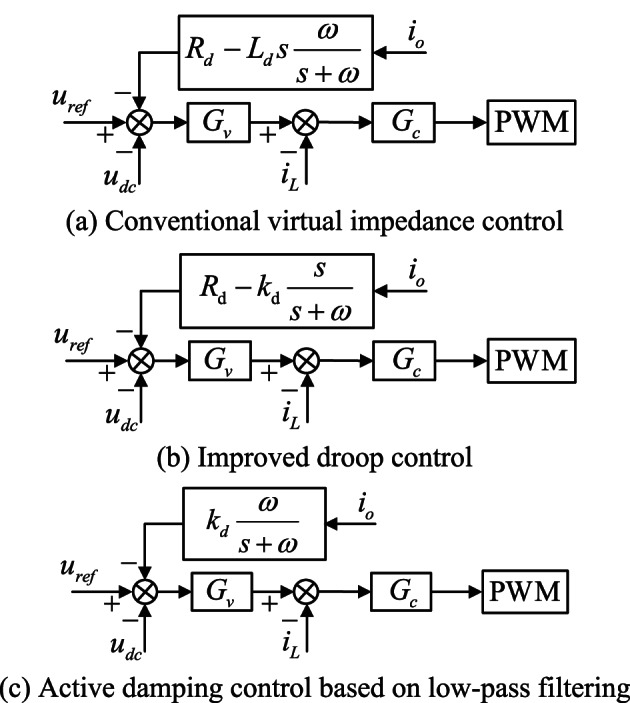
Several contrasting control methods

To contrast with the effect of the proposed control strategy, three common improved control strategies were selected for simulation comparison, and the bus voltage waveforms obtained are shown as (d)-(f) in Fig. 17.The graph also demonstrates the effectiveness of the proposed control method in damping oscillations and minimizing voltage fluctuations. Figure 17 (d) employs the traditional virtual impedance control strategy^25^. Figure 17 (e) adopts the improved droop control^26^, while Fig. 17 (f) showcases the active damping control based on low-pass filtering^27^. The control block diagrams for each of these methods are illustrated in Fig. 18.

**Fig. 19:**
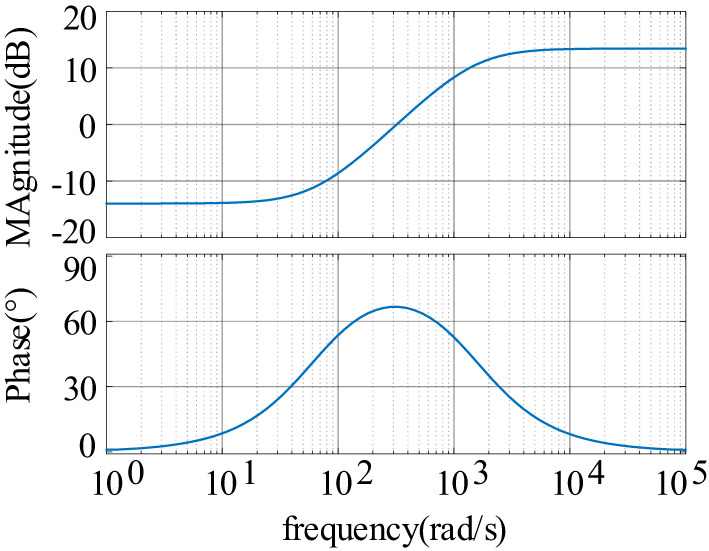
Bode diagram of virtual impedance is included for the droop loop

All these three control methods exhibited significant voltage drops during the voltage stabilization period, and their control effects were equivalent to inserting a virtual impedance. The impedance characteristics of the constant power unit ports near the oscillation frequency were reformed from the original negative impedance characteristics to positive resistance characteristics, without changing the impedance characteristics in other frequency bands. This enabled the system to effectively suppress bus voltage oscillations, maintain system stability, and at the same time, there was no large steady-state voltage deviation, effectively improving voltage quality, as shown in Fig. 19. Consequently, the voltage reference value varies with fluctuations in the converter output current. Notably, the control method demonstrates reduced damping efficacy when the load power is higher, as shown in Fig. 17(d)(e)(f) after t = 1s. Conversely, the control strategy introduced in this study solely impacts the current loop of the control system, maintaining a positive resistance only near the oscillation frequency without altering the low frequency range. It does not alter the original negative impedance characteristics of the low-frequency band. Therefore, it will not result in a significant voltage drop due to the presence of virtual positive resistance, leading to a more effective voltage stabilization.

**Fig. 20:**
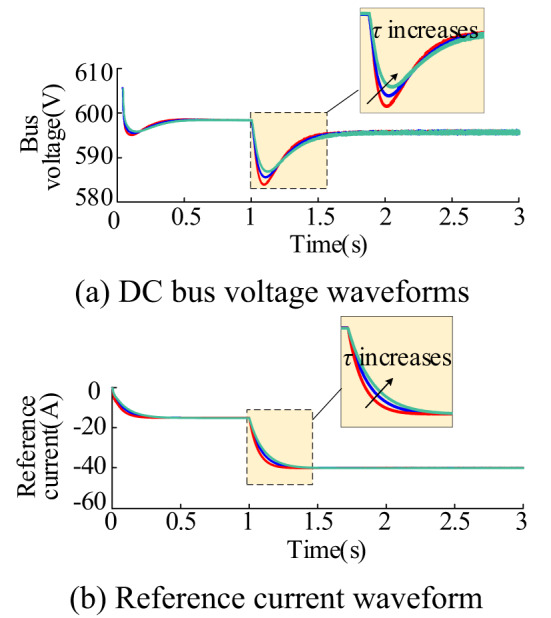
Voltage and current waveforms corresponding to different time constants *τ*

**Fig. 21:**
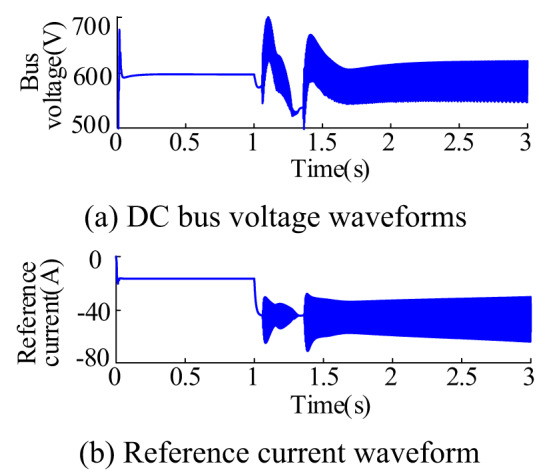
Time constant *τ* corresponds to voltage and current waveforms when improperly selected

Figure 20 shows the waveforms of bus voltage and current reference value $I_{{{\text{Lref}}}}^{*}$ under different time constants. The steady-state values of bus voltage and current reference remain essentially unchanged under different time constants. However, the dynamic response speeds vary, with response speeds decreasing and response times lengthening as the time constant *τ* increases. For different control methods, the *τ* value needs to meet various conditions to ensure that the constant power unit impedance near the oscillation frequency exhibits positive resistivity. When the *τ* value is too small to meet these conditions, the corresponding bus voltage waveform and current reference values are shown in Fig. 21. In the scenario where the conditions are not met (*τ* = 1e-3), the proposed control method cannot effectively maintain the system’s stability. In order to ensure the stability of the system while considering improved dynamic response performance, the appropriate time constant should be selected from Fig. 20(a). When the energy storage in the charging state of the system’s oscillation frequency is about 1458 rad/s, it is necessary to ensure that ${1 \mathord{\left/ {\vphantom {1 {\tau \ll 1458{\text{rad/s}}}}} \right. \kern-0pt} {\tau \ll 1458{\text{rad/s}}}}$. In practice, to ensure both system stability and quick response to system requirements simultaneously, the ratio 1/*τ* ≈(*ω*_res_/10). Therefore take the time constant can be set to 6e-3s. From Fig. 21(a), the oscillation frequency of the system is approximately 707 rad/s when the energy storage is in the discharging state, then $\sqrt {{{{k_{{\text{iu}}}}} \mathord{\left/ {\vphantom {{{k_{{\text{iu}}}}} \tau }} \right. \kern-0pt} \tau }} \ll 707{\text{rad/s}}$. In practice, to ensure system stability and response speed, it is recommended that $\sqrt {{{{k_{{\text{iu}}}}} \mathord{\left/ {\vphantom {{{k_{{\text{iu}}}}} \tau }} \right. \kern-0pt} \tau }}$ ≈(*ω*_res_/10). This implies a time constant of 8e-3s.

### Experimental verification

In order to further verify the effectiveness of the proposed control strategy, an experimental setup based on the RT-LAB semi-physical test platform is constructed for experimental study. The relevant circuit parameters and controller parameters are consistent with the simulation model.

**Fig. 22:**
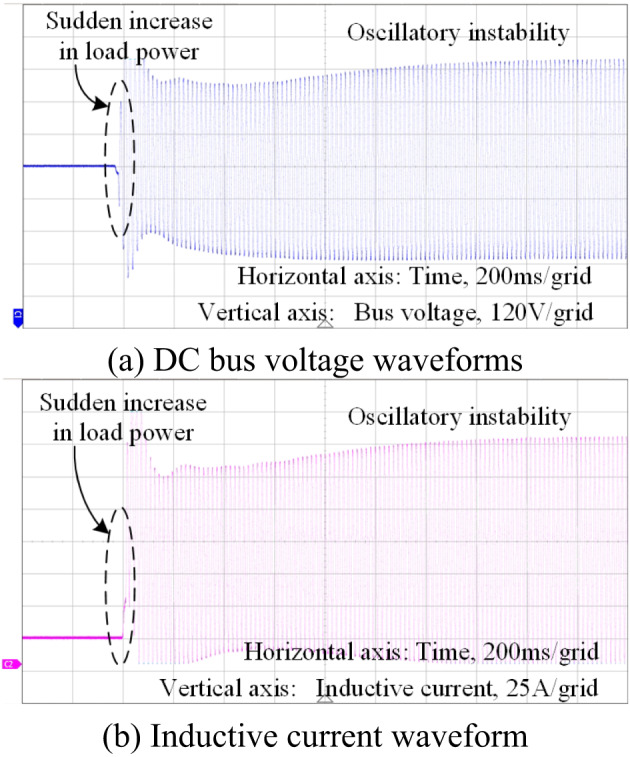
Voltage and current waveforms without introducing the proposed control method

When the energy storage is in the discharged state, without introducing the impedance shaping based feed-forward control method, the system bus voltage and the inductor current waveforms of the energy storage unit are shown in Fig. 22(a)(b). When a sudden increase in load power occurs (from 20 kW to 30 kW), both the voltage and current waveforms oscillate, leading to a loss of stability in the system.

**Fig. 23:**
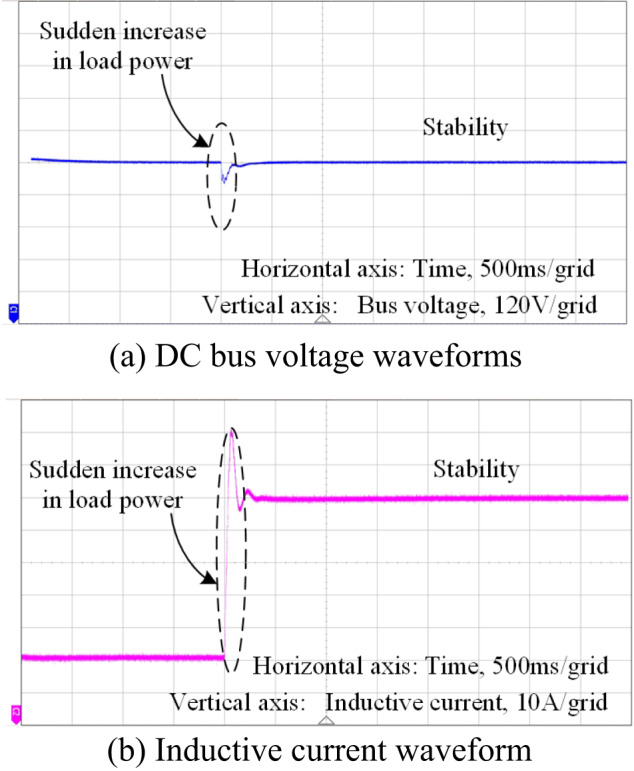
Voltage and current waveforms introducing the proposed control method(energy storage discharge state)

After the introduction of feed-forward control based on impedance shaping, the system bus voltage and inductor current waveforms are shown in Fig. 23(a)(b). The system voltage and current can maintain good stability during sudden changes in load power, with a small steady-state deviation of the bus voltage. The current waveforms can also track changes in the reference current value steadily during sudden increases in load power, ensuring that the system operates in a stable state consistently.

**Fig. 24:**
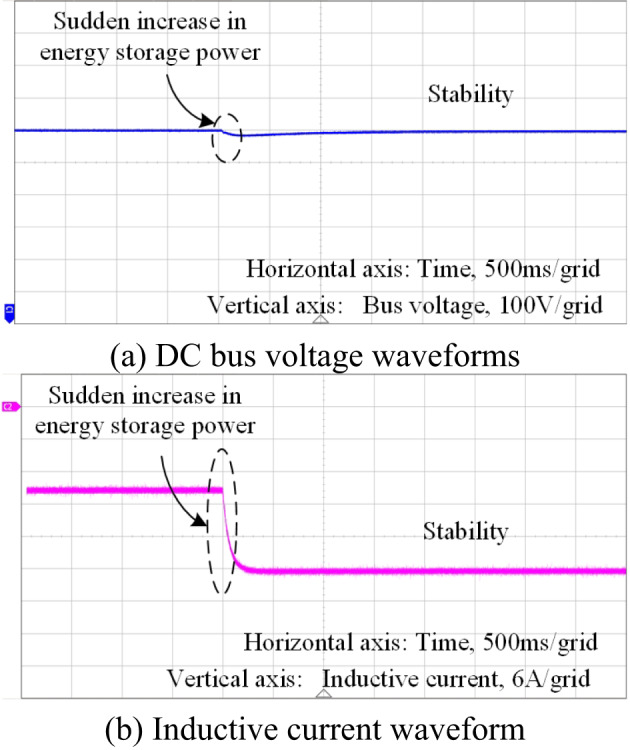
Voltage and current waveforms introducing the proposed control method(energy storage charge state)

When the energy storage is in the charged state, after implementing impedance shaping-based feed-forward control, the system bus voltage and inductor current waveforms are shown in Fig. 24(a)(b). The system voltage and current can maintain better stability during sudden changes in the power absorbed by the energy storage (from 8 kW to 20 kW). Additionally, the current waveforms can accurately track the required current values after the power change.

It can be seen that the experimentally verified waveforms and simulation results of energy storage operating in both charging and discharging states can maintain system stability. This verifies the effectiveness of the feed-forward control method based on impedance shaping.

## Conclusion

In this paper, considering that the energy storage unit under constant-current charging control presents negative impedance characteristics, which can lead to system oscillation and instability, a stabilization control method based on impedance shaping is proposed to improve system stability. The specific conclusions are as follows:

- (a) The impedance of the energy storage unit port is shaped by creating a current feed-forward compensation transfer function, which ensures that the reference current tracks the bus voltage. The feed-forward compensation transfer function has only one parameter, namely the time constant. This feature makes the proposed control method simpler and eliminates the necessity to adjust the existing controller parameters.
- (b) When the time constant meets specific conditions, the port impedance of the constant power unit shows a positive resistive characteristic near the oscillation frequency. This enhances the phase margin of the system and promotes stable system operation. Simultaneously, the original negative impedance characteristic is retained in the low-frequency band, preventing significant voltage drops in the system.
- (c) The small signal analysis is used to determine the suitable controller parameters and the range of the time constant values. Simultaneously, it validates the effectiveness of the proposed control method. Through simulation and experimentation, the accuracy of the analysis results is verified. The proposed control method can stabilize the DC bus voltage, enhance system stability, and prevent significant voltage dips.

## Data Availability

All the data generated or analyzed during this study are public and included in this published article.

## References

[1] Yang, J. et al. Distributed cooperative control method and its application in power system. *Trans. China Electrotechnical Soc.* **36** (19), 4035–4049 (2021).

[2] Hassan, M. A. et al. Jan., DC Microgrids for Renewable Energy Integration: Architectures and Control Strategies, IEEE Transactions on Sustainable Energy, vol. 14, no. 1, pp. 310–325, (2023).

[3] Cheng, L. et al. Review and prospect on operational reliability of power distribution system with multiple distributed resources. *Autom. Electr. Power Syst.* **45** (22), 191–207 (2021).

[4] Li, Y. & Wu, W. Advanced control strategies for PV-Storage DC microgrids in renewable energy applications. *IEEE J. Emerg. Sel. Top. Power Electron.* **11** (2), 1896–1910 (2023).

[5] Zhang, Q. et al. Active damping suppression strategy of bus voltage Oscillation in Source-charge cascade system of marine microgrid. *J. Power Syst. Autom.* **34** (3), 1–10 (2022).

[6] Wu, X. et al. Research progress and prospect of broadband Oscillation analysis and suppression in microgrid (swarm). *Power Grid Technol.* **47** (9), 3727–3745 (2023).

[7] Liu, Z., Wu, X. & Filter-Induced, L. C. Oscillations in DC Microgrids: Frequency-Domain Modeling and Stability Criteria, IEEE Transactions on Smart Grid, vol. 14, no. 5, pp. 3568–3582, Sept. (2023).

[8] Han, L. et al. Feb., Real-Time Oscillation Detection in DC Microgrids Using Wideband Impedance Spectroscopy, IEEE Transactions on Industrial Informatics, vol. 20, no. 2, pp. 1789–1801, (2024).

[9] Zhang, H. et al. Comparative analysis of impedance criterion and admittance criterion for DC distribution system. *Power Grid Technol.* **45** (3), 1167–1174 (2021).

[10] Gu, Y. et al. Sept., Extended Nyquist Stability Criterion for AC-DC Hybrid Microgrids with Multiple Voltage Source Converters, IEEE Transactions on Power Systems, vol. 38, no. 5, pp. 4123–4137, (2023).

[11] Liu, P. et al. Research on unified analysis method of Grid-connected converter stability based on impedance analysis. *Power Syst. Prot. Control*. **51** (4), 114–125 (2023).

[12] Jia, K. et al. Resonant suppression strategy and passive damping selection method of photovoltaic grid-connected system. *Autom. Electr. Power Syst.* **45** (15), 109–114 (2021).

[13] Zhang, H. et al. Stability analysis of DC microgrid based on passive damping. *High. Voltage Technol.* **43** (9), 3100–3109 (2017).

[14] Ming, J. et al. Large signal stability analysis and active damping compensation method of DC microgrid. *Trans. China Electrotechnical Soc.* **36** (S2), 517–529 (2021).

[15] You, X. et al. Stability analysis and active damping method of bipolar DC system with constant power load. *Trans. China Electrotechnical Soc.* **37** (4), 918–930 (2022).

[16] Zhuang, X. et al. Active damping control strategy for constant power load of ship All-electric propulsion system. *Trans. China Electrotechnical Soc.* **35** (S1), 101–109 (2020).

[17] Zhang, X., Ruan, X. & Zhong, Q. Improving the stability of cascaded DC/DC converter systems via shaping the input impedance of the load converter with a parallel or series virtual impedance. *IEEE Trans. Industr. Electron.*, **62**, 12, pp. 7499–7512, 2015.

[18] Guan, Y. et al. The dual-current control strategy of grid-connected inverter with LCL filter[J]. *IEEE Trans. Power Electron.* **34** (6), 5940–5952 (2018).

[19] Zeng C, Zhao Y, Miao H, et al. Active damping strategy for two-stage grid-connected converters with PV output fluctuations under weak grid conditions[J]. Journal of Power Electronics, 2025: 1-12.

[20] Zhu, X. & Meng, X. Stability analysis and active damping control of DC microgrid. *High. Voltage Technol.* **46** (5), 1670–1681 (2020).

[21] Hosseinipour & Hojabri, H. Small-signal stability analysis and active damping control of DC microgrids integrated with distributed electric springs. *IEEE Trans. Smart Grid*. **11** (5), 3737–3747 (2020).

[22] Oshnoei, M. et al. Adaptive damping control to enhance Small-Signal stability of DC microgrids. *IEEE J. Emerg. Sel. Top. Power Electron.* **11** (3), 2963–2978 (2023).

[23] Yoo A, Lee W J, Dehkordi B M, et al. Input filter analysis and resonance suppression control for electrolytic capacitor-less inverter[C]//2009 Twenty-Fourth Annual IEEE Applied Power Electronics Conference and Exposition. IEEE, 2009: 1786-1792.

[24] Shin H, Ha J I, Lee W J. Active power control for minimum switching of three-phase electrolytic capacitor-less PWM converter[C]//2013 IEEE Energy Conversion Congress and Exposition. IEEE, 2013: 783-788.

[25] Guo Li, F. & Yibin, L. Analysis of DC microgrid stability and damping control methods [J]. *J. Electr. Eng. China*. **36** (04), 927–936 (2016).

[26] Zhi Na, Z. & Hui, X. Research on improved droop control strategy for improving the dynamic characteristics of DC microgrids [J]. *J. Electr. Eng.* **31** (03), 31–39 (2016).

[27] Zhang Qinjin, Z. et al. Active damping strategy for bus voltage Oscillation in Source-Load cascade systems of ship microgrids [J]. *J. Electr. Power Syst. Autom.* **34** (03), 1–10 (2022).

